# Comparison of topical hypericum perforatum and metformin effectiveness in wound healing in streptozotocin-induced diabetic rats

**DOI:** 10.3389/fendo.2025.1691294

**Published:** 2025-11-05

**Authors:** Banu Turhan, Sönmez Sağlam, Mücahit Osman Yücel, Raşit Emin Dalaslan, Mehmet Ali Sungur, Fatih Demir, Zekeriya Okan Karaduman, Mehmet Arıcan

**Affiliations:** ^1^ Department of Pediatric Endocrinology, Atatürk Sanatoryum Education and Research Hospital, Ankara, Türkiye; ^2^ Department of Orthopedics and Traumatology, Düzce University Medical Faculty, Düzce, Türkiye; ^3^ Department of Biostatistics and Medical Informatics, Düzce University Medical Faculty, Düzce, Türkiye; ^4^ Department of Pathology, Düzce University Medical Faculty, Düzce, Türkiye

**Keywords:** diabetes mellitus, *Hypericum perforatum*, metformin, rat, wound healing

## Abstract

**Purpose:**

Our study aimed to assess and compare the effectiveness of topically applied Hypericum perforatum (HP) and metformin on wound healing in diabetic rats.

**Methods:**

Thirty-two male Wistar rats (8–10 weeks old, 250 ± 50g) were divided into four groups of eight rats each: Control, Diabetes mellitus (DM), Metformin (Met), and HP. Diabetes was induced in all experimental rats except the control group using streptozotocin (STZ) (60mg/kg, intraperitoneal). A full-thickness skin defect was created in all rats. Two milliliters (ml) of sterile saline were administered to the Control and DM groups, two ml of metformin (10% gel, topical) to the Met group, and two ml of HP (olive oil extract) to the HP group, repeated every 24 hours for 14 days. The condition of the lesions was monitored on days 0, 3, 7, 10, and 14, and the extent of contraction and granulation tissue formation was documented. At the 14^th^ day, the lesioned areas were examined histopathologically and immunohistochemically.

**Results:**

The baseline characteristics of the rats before the study showed no significant differences between the groups (p>0.05). The HP group had the smallest final wound size and the highest wound contraction percentages from day 0 to 14 (p<0.001 for both). There was no statistically significant difference among the groups in the collagen index (p=0.118). The fibroblast density scores in the DM group were significantly lower than those in the other groups (p=0.004). The hypertrophic index values of the HP group were the highest compared to the other groups (p=0.003). Although the HP and control groups exhibited higher TGF-β percentages and H scores than the other groups, these differences were not statistically significant (p=0.660 and p=0.647).

**Conclusion:**

Topically applied HP in uncontrolled DM rats improved wound healing scores more than the non-diabetic controls. Metformin also significantly enhanced healing in DM rats, with results comparable to controls. Since HP and metformin are easily accessible, further research could lead to cost-effective treatments for wound healing issues in DM patients.

## 1 Introduction

Diabetes mellitus (DM) is a widespread disease worldwide, and wound healing complications occur in all patients with DM, regardless of the subtype. The underlying mechanisms of these wound healing problems include impaired circulation, reduced immunity, and an imbalance in the local wound healing process (1). Although wound issues are often linked to diabetic foot ulcers in patients with long-term DM, DM-related skin and wound healing problems are also common in children (2). Particularly in patients with uncontrolled DM, consistently high blood glucose levels can disrupt tissue oxygenation and potentially cause wound healing problems during the acute phase (1). The global prevalence of diabetes in 2021 was estimated at 10.5% (536.6 million individuals), projected to increase to 12.2% (783.2 million individuals) by 2045 (3). Given that the incidence of type 1 DM is rising and the age at first diagnosis has decreased to under 4 years, it is evident that more children are likely to face DM-related complications at a young age (4). With advancements in technology and increased access, more children with DM are using devices like continuous glucose monitors and insulin pumps, which can lead to skin and wound issues associated with prolonged device use (5).

In cases of wound complications observed in patients with DM, local wound management is equally crucial as systemic regulation of DM. *Hypericum perforatum L.* (HP) (St. John’s Wort) extract, which has been utilized as a traditional remedy for numerous years, is a substance that enhances efficacy in instances where skin integrity is compromised, such as ulcers and burns (6). HP has the capacity to expedite wound healing by inhibiting the heightened inflammatory response characteristic of diabetic environments and facilitating the migration of fibroblasts. Additionally, it mitigates the detrimental effects of oxidative stress on wound tissue owing to its antioxidant properties (6, 7). HP’s active components include phloroglucinol derivatives (mainly hyperforin at 0.2%–4%), naphtodianthrones (hypericin, isohypericin, pseudohypericin at 0.1%–0.15%), flavonoids (rutin 1.6%, hyperoside 0.9%, isoquercitrin 0.3%), tannins (8%–9%), and essential oils (0.05%–0.9%) (8). Recent research has concentrated on the utilization of cost-effective and widely available herbal extracts in the process of wound healing (9). Experimental studies conducted *in vitro* and *in vivo* have demonstrated that olive oil macerate of the HP plant reduces the inflammation period, promotes fibroblast migration, and enhances collagen deposition, thereby expediting the wound healing process (10). Furthermore, the literature includes not only experimental research but also some clinical studies investigating the effects of HP on burn and wound management (11, 12). The biological effects of HP are derived from its active constituents, which include hyperforin, hypericin, flavonoids, and tannins. However, the concentration of these constituents may vary depending on the geographic region where the plant is cultivated, the extraction methodology employed, and the type of solvent utilized. As a result, phytochemical variability complicates the comparison of results across different studies (6–8).

Metformin is taken orally as the main treatment to control blood glucose levels in patients with type 2 DM. Metformin reduces glucose levels by decreasing hepatic glucose production (gluconeogenesis), limiting glucose absorption in the intestines, and increasing peripheral glucose uptake and use, thereby enhancing insulin sensitivity (13). Beyond its systemic application, several experimental studies documented in the literature suggest that metformin may also have localized effects in facilitating wound healing (14). Among the systemic effects of this biguanide agent, it has been reported to augment activation of AMP-activated protein kinase, thereby enhancing vascular endothelial function during the wound-healing process (15). Locally, evidence indicates that metformin contributes to wound repair by promoting angiogenesis, re-epithelialization, and collagen deposition (16, 17). Furthermore, some *in vivo* experimental studies have reported that metformin may impede wound healing by inducing cell cycle arrest and reducing cellular proliferation (18). The evaluation of metformin in topical formulation is predicated on the hypothesis that it may exert efficacy directly within the wound microenvironment, extending beyond its systemic role in glycemic regulation. Consequently, the potential to promote angiogenesis, epithelialization, and collagen synthesis locally, independent of systemic adverse effects, can be explored. This methodology also represents a strategic approach to repositioning existing pharmaceuticals for wound healing applications.

Few studies have investigated the effects of metformin and HP on wound healing, and none in the English literature directly compares these two active ingredients. This study was conducted to assess and compare the effectiveness of locally applied HP oil extract and metformin gel in wound healing, using a streptozotocin (STZ)-induced diabetic rat wound model.

## 2 Materials and methods

### 2.1 Animals

The study was conducted following the guidelines of the Helsinki Declaration and was approved by the Düzce University Animal Experiments Local Ethics Committee (protocol code 03/20/2024, approval date: March 20, 2024). The male Wistar rats (n = 32) used in this study were obtained from the Düzce University Animal Research and Application Center. The rats were approximately 8–10 weeks old, weighed 250 ± 50 g, and were monitored for any signs of health issues during a 15-day adaptation period. They were housed in polycarbonate cages in a temperature-controlled room (22–24 °C) with a 12-hour light/dark cycle. Standard pellets and water were provided ad libitum. The standard diet utilized comprises approximately 20% protein, 5% fat, and 55% carbohydrates.

The rats were assigned to four groups through simple randomization, with eight rats in each group.

*Control:* Non-diabetic group, only sterile saline (SS) was applied to the wound.
*DM*: Diabetic group, only SS was applied to the wound.
*Met*: Diabetic group, topical *metformin* gel was applied to the wound.
*HP:* Diabetic group, topical HP was applied to the wound.

### 2.2 Diabetes induction

At the beginning of the study, the rats’ blood glucose levels, weights, and body lengths (from the nose to the anus and from the nose to the tail end) were measured and recorded.

Diabetes was induced in the rats of the experimental group with a single intraperitoneal (IP) dose of 60 mg/kg STZ (Glentham, England) in citrate buffer solution (0.1 M, pH 4.5), prepared immediately before administration (19, 20). After one week, blood samples were collected from the tail vein and analyzed for fasting glucose levels using a glucometer (Accu-Check, Roche). Rats with blood glucose levels exceeding approximately 250 mg/dL were considered diabetic and selected for further experiments. Rats with blood glucose below this threshold were excluded from the study (21).

### 2.3 Chemicals

*Hypericum perforatum extract*. To enhance the local effects, an olive oil extract of HP was prepared using a well-established protocol (10). The aerial parts of HP were collected from the plateaus of Düzce in June 2024. 25 grams of fresh HP flowers were placed in a glass jar containing 250 ml of olive oil and kept for 4 weeks. The mixture was maintained at room temperature (25-35 °C) during the waiting period and exposed to sunlight for 12 hours a day (7). The Hypericum perforatum olive oil extract used in the study contains the primary active components described in the literature (hyperforin, hypericin, flavonoids, tannins), and the levels of these components may vary depending on environmental and technical factors (8).

*Metformin hydrochloride topical gel*. Metformin powder was procured from “Sigma Aldrich,” and a 10% metformin gel was formulated. The 10% metformin gel dosage employed is grounded in concentrations demonstrated to be effective in the literature. This formulation has been documented to deliver a potent local effect with minimal systemic absorption (22).

### 2.4 Excisional wound model

The anesthetic dosage administered to the rats was established by weighing each subject using an electronic scale. A combination of 50 mg/kg ketamine (Eczacıbaşı, Istanbul, Turkey) and 10 mg/kg xylazine (Bayer, Istanbul, Turkey) was administered via intramuscular injection into the left groin region of the rats (23). After anesthesia, the rats’ backs were shaved and prepared with a 10% Batticon solution (Batticon, Adeka, Turkey). A full-thickness skin defect was created in all rats using a surgical blade.

Two milliliters (ml) of SS were applied to the Control and DM groups, two ml of metformin to the Met group, and two ml of HP to the HP group, forming a thin layer on the wound (24). This treatment protocol was administered to all groups every 24 hours for 14 days until the study’s conclusion. No dressings were used on any groups to maintain traditional practices and prevent self-cannibalization. After surgical procedures, rats received fentanyl citrate (Polifarma, Tekirdağ, Turkey) at a dose of 0.02 mg/kg subcutaneously for three days to manage pain. A veterinarian specialist monitored the rats, with four animals housed per cage. Blood glucose levels were measured weekly in the diabetic groups, with the aim of excluding rats whose blood glucose levels fell below the target diabetic range from the study.

**Figure 1:**
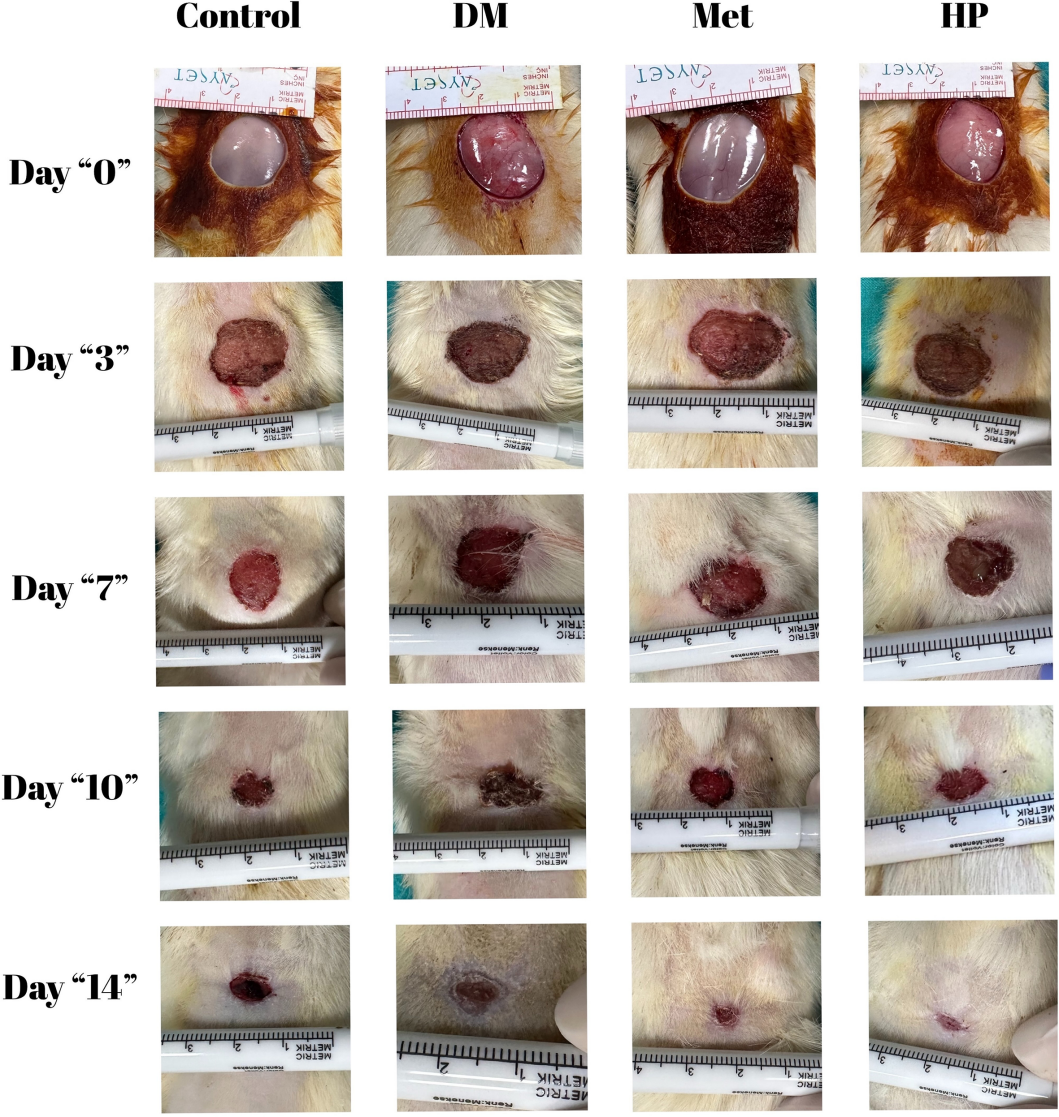
Scar size changes of the groups during the study period.


Skin lesions were documented on days 0, 3, 7, 10, and 14 using a digital camera and a reference length measurement unit (**Figure 1**). Wound areas (mm^2^) were measured with ImageJ software (NIH, USA), and wound shrinkage over time was calculated. Additionally, the contraction rate of the wound size during the 14 days was expressed as a percentage of the initial wound size (6):

$$Wound\;closure\;percentage=\frac{Original\;wound\;area-Current\;wound\;area}{Original\;wound\;area}x100$$

### 2.5 Histopathological analyses

**Figure 2:**
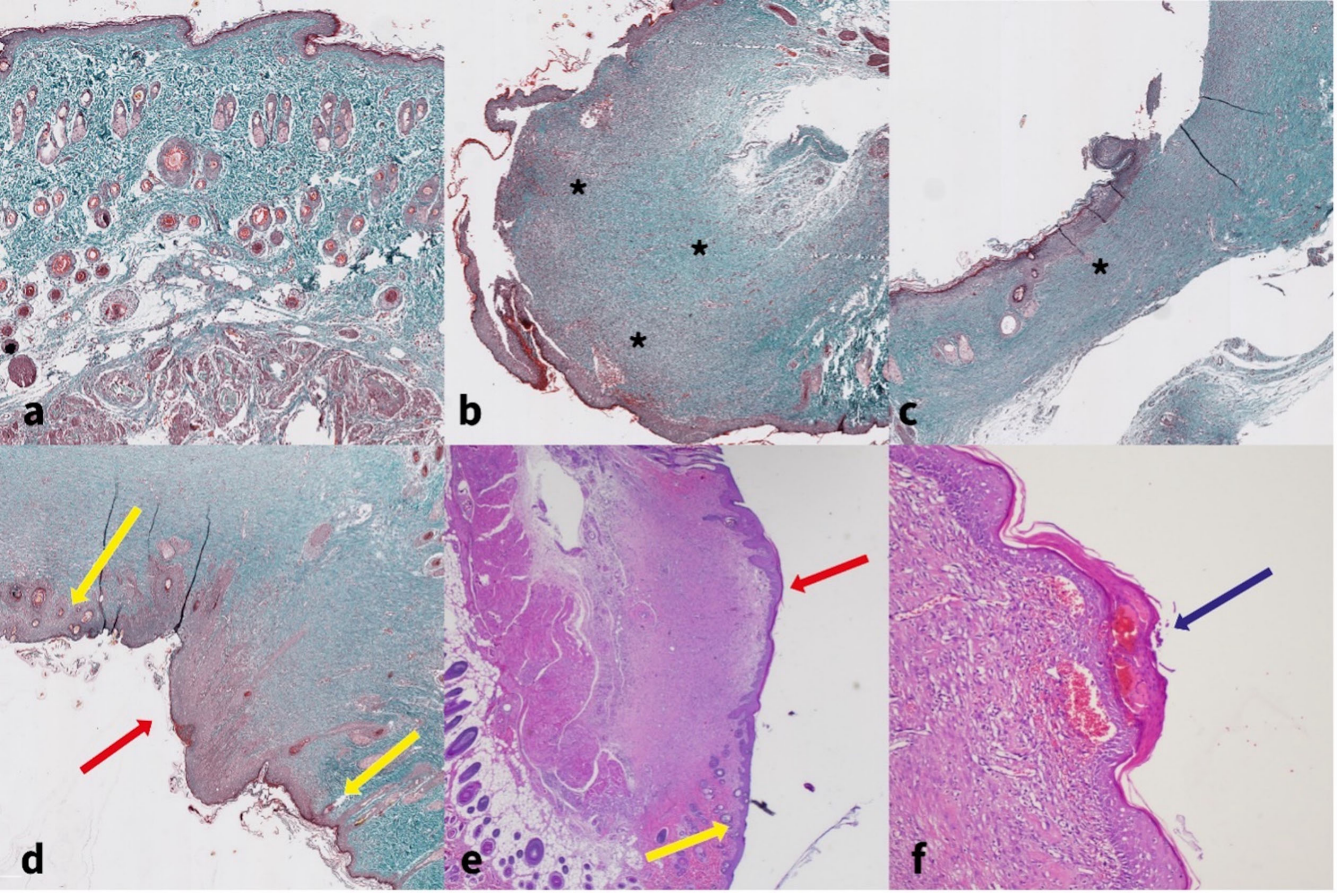
**(a)** Normal skin with preserved skin appendages, Masson’s Trichrome Staining. **(b)** Hypertrophic scar tissue (black asterisk) with incomplete epidermal reepithelialization. The epidermis appears mildly thickened compared to uninjured skin, Masson’s Trichrome Staining. **(c)** Hypoplastic scar tissue compared to uninjured skin, with incomplete reepithelization on the wounded epidermis, Masson’s Trichrome Staining. **(d)** Thickened, complete epidermis over scar tissue (red arrow) compared to normal skin epidermis (yellow arrow). **(e)** Nearly normal thickness of epidermis over hypertrophic scar tissue, H&E, 40x. **(f)** Parakeratotic hyperkeratosis over completely healed epidermis, H&E x100.

On day 14, all the animals were euthanized via an intraperitoneal overdose of sodium pentobarbital (Narcoren-Rhone Merienx) at a dosage of 150 mg/kg (23). The animals’ death was confirmed through intracardiac puncture (25). Skin samples, including the wound margin, were excised and fixed in 10% formalin, embedded in paraffin, and sectioned at 4–5 μm. Histological analysis used Hematoxylin and Eosin (H&E) for tissue structure and inflammation, and Masson’s Trichrome for collagen assessment (**Figure 2**). H&E staining involved dewaxing formalin-fixed, paraffin-embedded tissue sections in xylene and rehydrating through descending grades of ethanol to distilled water. The sections were stained with Harris hematoxylin for an appropriate time and washed in running tap water until nuclei appeared blue. Differentiation was performed in 1% acid alcohol (1% HCl in 70% ethanol) for 5–10 seconds, followed by rinsing in tap water until sections regained a blue color. Bluing was achieved by immersion in an alkaline solution and subsequent washing in tap water for 5 minutes. Counterstaining was performed with 1% eosin Y for 10 minutes. The sections were then rinsed in tap water for 1–5 minutes, dehydrated through ascending grades of alcohol, cleared in xylene, and mounted with a resinous medium (26). Morphometric analysis was conducted on the stained sections using both light microscopy and digital slide scanning. Slides were scanned using a whole-slide imaging (WSI) system to produce high-resolution digital images for quantitative analysis.

**Figure 3:**
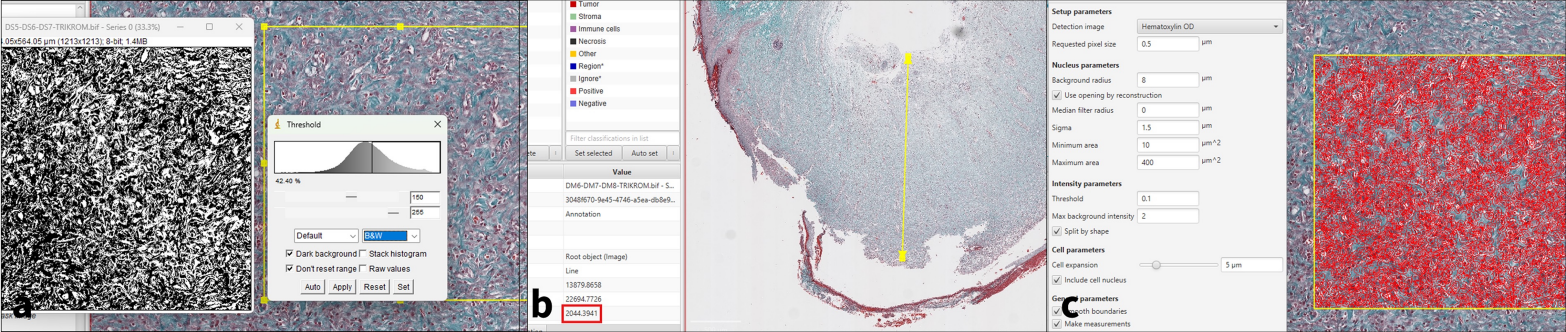
**(a)** Measuring collagen index with threshold function on ImageJ software. **(b)** Measuring scar tissue thickness and hypertrophic index on scanned image using Masson’s Trichrome staining. **(c)** Measuring fibroblast count on digital scanned image using QuPath software.

For the histological evaluation of wound healing, the SPOT scoring system (27) was used. This system assesses healing across six categories representing different stages of wound repair. The total SPOT score, obtained by summing the individual category scores, ranges from 0 to 12, with a score of 12 indicating complete healing. In addition to the SPOT scoring system, the previously described hypertrophic index (HI) and collagen index (CI) (28), as well as fibroblast density per 1 mm², were measured (**Figure 3**).

### 2.6 Immunohistochemistry

**Figure 4:**
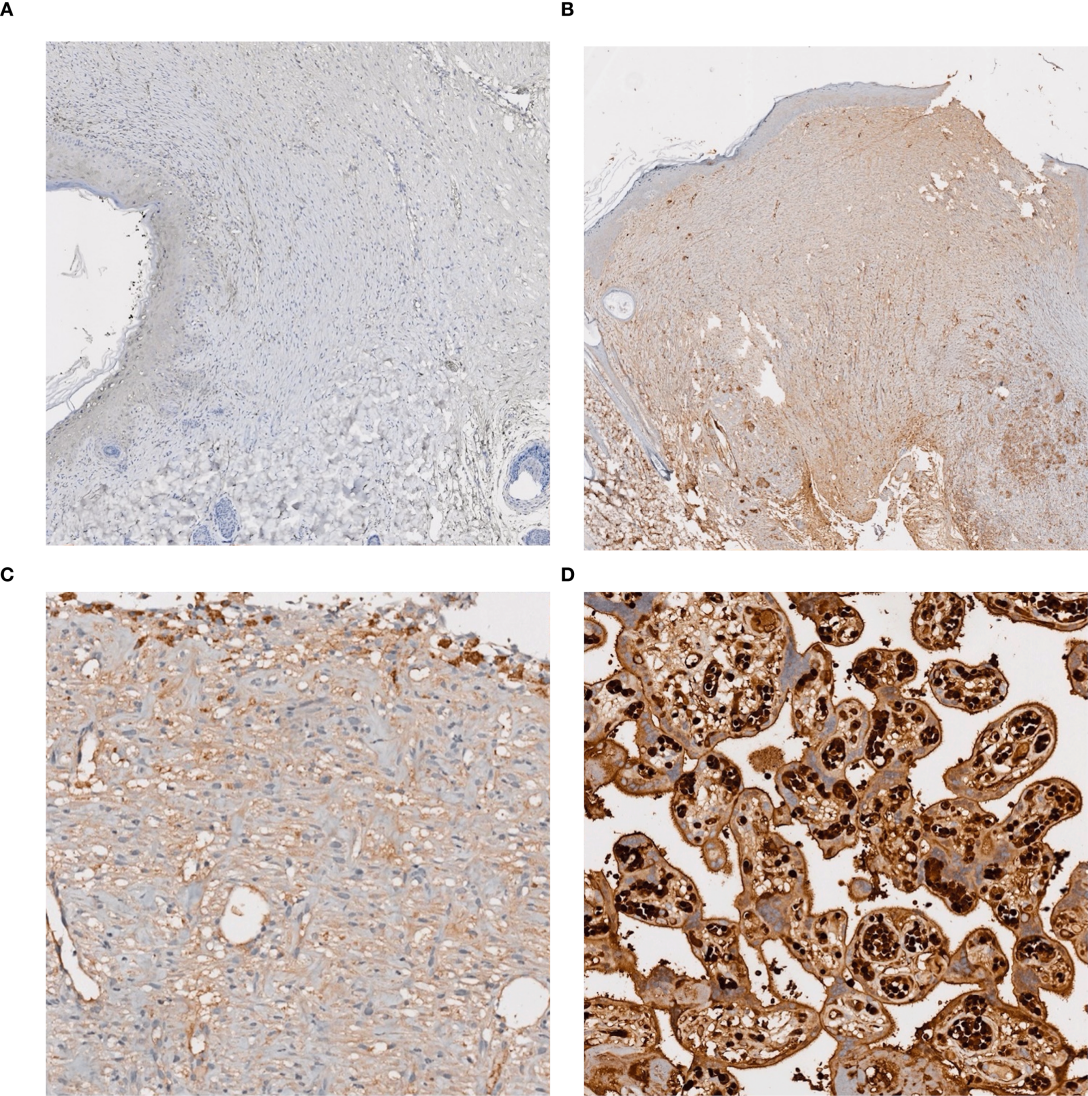
Representative immunohistochemical staining with TGF-β1. **(a)** Mostly negative expression with TGFβ1 in scar tissue, **(b)** Weak but diffuse staining with TGFβ1 in scar tissue, scanned image, low power field view, **(c)** Weak but diffuse staining with TGFβ1 in scar tissue, scanned image, high power field view, **(d)** Staining of TGFβ1 in placental control tissue.


To evaluate the molecular participation in the wound healing process, immunohistochemical staining for TGF-β1 (Santa Cruz, 1:100 dilution) was performed on paraffin-embedded sections (**Figure 4**). The sections were blocked following antigen retrieval and subsequently incubated with the primary antibody at room temperature for 32 minutes. They were then rinsed with Phosphate-Buffered Saline (PBS), and the secondary antibody was applied. Although the staining intensity was predominantly weak, certain cases demonstrated extensive expression. To ensure objectivity, the staining was assessed utilizing the H-score method as delineated by McCarty et al. (29). In summary, H-scores were determined employing the following formula:

$$\begin{array}{c} H-score\;=\;1\;\times \;\left(\%\;weak\right)\;+\;2\;\\ \times \;\left(\%\;moderate\right)\;+\;3\;\times \;\left(\%\;strong\right),\;with\;a\;possible\;range\;from\;0\;to\;300. \end{array}$$

### 2.7 Statistical analysis

IBM SPSS Statistics v.22 (IBM Corp., 2013, Armonk, NY, USA) was used as the statistical package for analysis. Before conducting inferential analyses, the assumption of normality was assessed using the Shapiro-Wilk test, and homogeneity of variances across groups was examined using Levene’s test. Between-group differences were evaluated using one-way analysis of variance (ANOVA), followed by an LSD *post hoc* test for pairwise comparisons. Given the within-subject design involving repeated measurements, repeated measures ANOVA was employed to examine changes over time and potential time-by-group interactions. Bonferroni and LSD tests were used for multiple comparisons and within-group modifications when necessary. The Fisher-Freeman-Halton test, along with a Bonferroni-adjusted Z-test for comparing column proportions, was employed in the analysis of categorical data. A p-value of less than 0.05 was considered statistically significant throughout all analyses.

## 3 Results

The baseline characteristics of the rats prior to the study revealed no significant differences between the groups; on day 0, all rats exhibited similar blood glucose levels, lengths, and weights (p > 0.05). The distribution of these measurements before the study is detailed in **Table 1**.

**Table 1:** Distribution of animals’ height, weight, and blood glucose levels before the study.

Parameter	Control	DM	Met	HP	p
Length (Nose to Anus in cm)	18.38 ± 0.92	18.63 ± 1.06	19.38 ± 0.74	19.00 ± 0.93	0.167
Length (Nose to tail end in cm)	36.00 ± 1.77	37.38 ± 1.85	37.75 ± 1.67	37.75 ± 2.49	0.257
Weight (g)	249.38 ± 6.41	248.63 ± 7.31	250.00 ± 4.50	249.00 ± 6.48	0.975
Initial Blood glucose (mg/dl)	77.38 ± 5.78	78.63 ± 5.99	77.50 ± 6.69	77.38 ± 3.70	0.964

DM, Diabetes mellitus; Met, Metformin; HP, Hypericum perforatum; cm, centimeters; mg, milligrams; dl, deciliters; g; grams.

**Figure 5:**
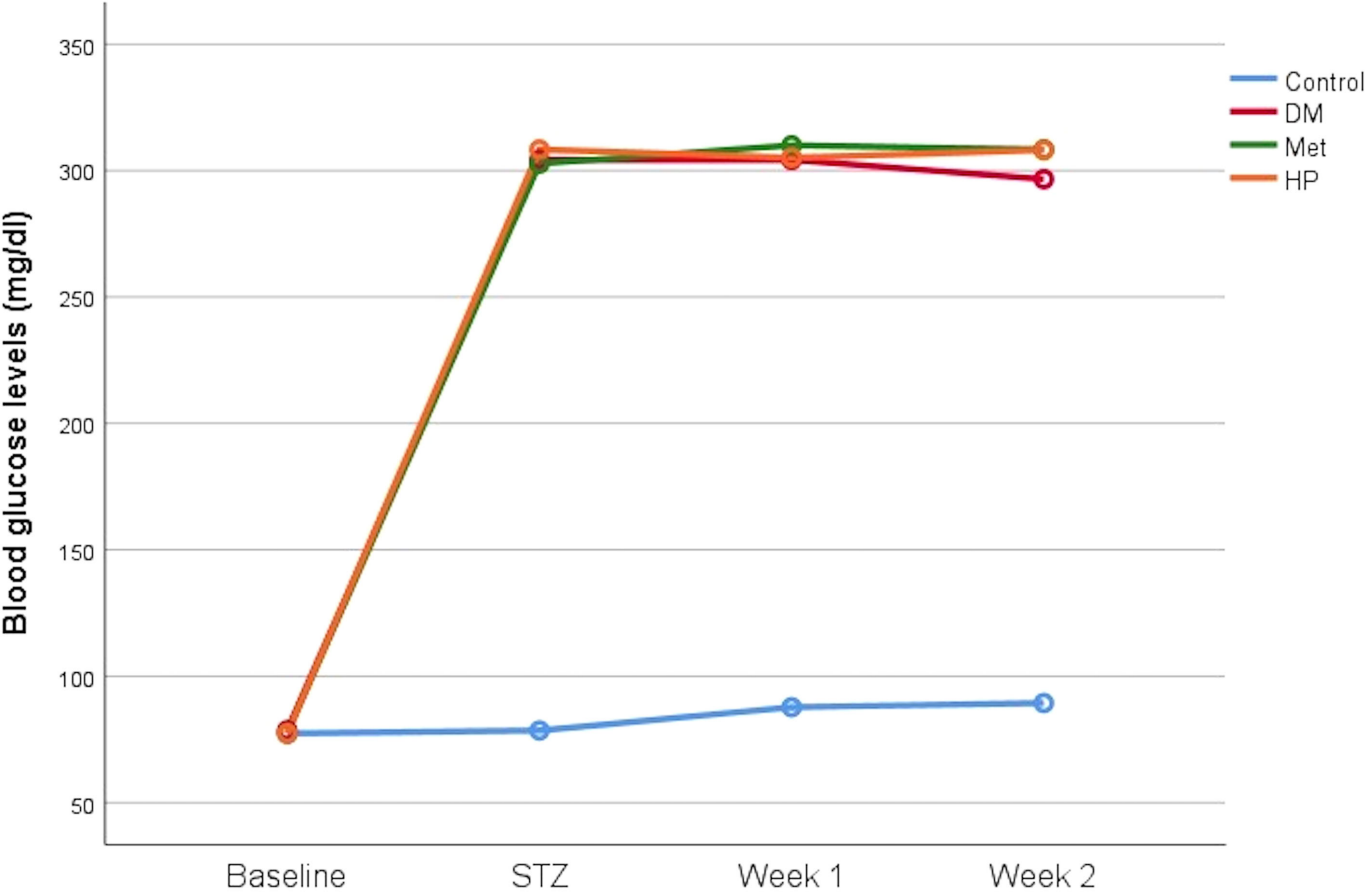
Changes in blood glucose levels across the groups during the study period.

Analysis of blood glucose levels before and during treatment revealed significant differences between the control group and the diabetic groups induced by STZ (p<0.001). All three DM-induced groups showed significant increases after STZ injection, as indicated by the comparison of pre- and post-injection values. However, there were no statistically significant differences between the DM, Met, and HP groups after the STZ injection (p>0.05). Even the control group, which did not receive STZ, experienced significant increases in blood glucose levels during the first and second weeks of treatment (p< 0.05) (**Table 2**, **Figure 5**).

**Table 2:** Mean and standard deviations (SD) of rats’ blood glucose levels during the study period.

Blood glucose levels (mg/dl)	Control	DM	Met	HP	p
Baseline	77.38 ± 5.78	78.63 ± 5.99	77.50 ± 6.69	77.38 ± 3.70	0.964
STZ	78.50 ± 6.66^a^	304.25 ± 19.16^b^	302.75 ± 23.16^b^	308.38 ± 18.56^b^	**<0.001**
Week 1	87.75 ± 3.06^a^	304.25 ± 19.16^b^	310.00 ± 18.21^b^	305.13 ± 16.38^b^	**<0.001**
Week 2	89.38 ± 4.34^a^	296.63 ± 12.11^b^	308.38 ± 18.58^b^	308.13 ± 17.39^b^	**<0.001**
p_0-S_	0.610	**<0.001**	**<0.001**	**<0.001**	
p_0-1_	**0.003**	**<0.001**	**<0.001**	**<0.001**	
p_0-2_	**0.001**	**<0.001**	**<0.001**	**<0.001**	
p_S-1_	**0.012**	0.999	0.531	0.736	
p_S-2_	**0.009**	0.215	0.282	0.977	
p_1-2_	0.116	0.460	0.889	0.685	

0: Baseline, S: STZ, 1: Week 1, 2: Week 2.

^a,b^Different superscript letters denote significant differences between the groups in each measurement time according to the post hoc test.

STZ: 7 days after the STZ application, mg; milligrams, dl; deciliters.

DM; Diabetes mellitus, Met; Metformin, HP; Hypericum perforatum.

Bold values indicate statistically significant differences (p<0.05).

Throughout the study, the diabetic groups consistently weighed less than the control group during the first and second weeks (p=0.007 and p<0.001, respectively). In the control group, weight declined during the first week of treatment and then increased by the end of the second week (p<0.001). Conversely, in the three diabetic groups, weight also decreased initially. Then it increased in the second week, but these weights remained lower than those of the control group (p<0.001) (**Table 3**).

**Table 3:** Mean and standard deviations (SD) of rats’ weights during the study period.

Weight (g)	Control	DM	Met	HP	p
Baseline	249.38 ± 6.41	248.63 ± 7.31	250.00 ± 4.50	249.00 ± 6.48	0.975
Week 1	241.88 ± 6.20^a^	230.75 ± 8.84^b^	233.00 ± 6.16^b^	229.75 ± 6.76^b^	**0.007**
Week 2	276.50 ± 6.35^a^	244.13 ± 7.20^b^	243.50 ± 4.99^b^	243.38 ± 7.07^b^	**<0.001**

P< 0.001 for all pairwise comparisons across experimental days within each group.

^a,b^Different superscript letters denote significant differences between the groups in each measurement time according to the post hoc test.

DM; Diabetes mellitus, Met; Metformin, HP; Hypericum perforatum, g; grams.

Bold values indicate statistically significant differences (p<0.05).

**Figure 6:**
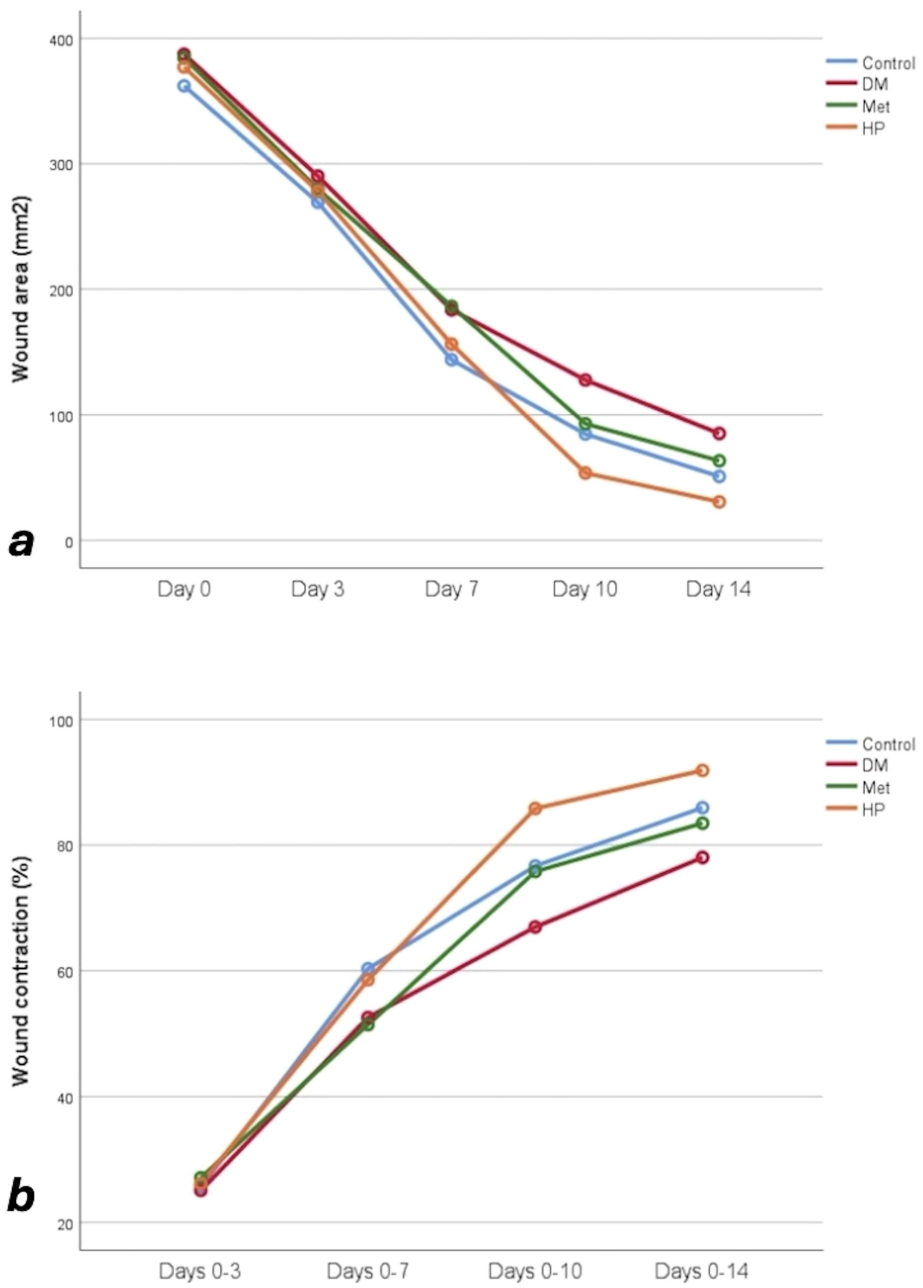
**(a)** Wound area measurements of the study groups over the course of the study. **(b)** Wound contraction rates (%) among the study groups.

The wound size measurements across the groups showed significant changes during the study period; all groups experienced notable reductions from day 0 to 14 (p<0.001). On day 3, the DM group had the largest wound size (p=0.030). By day 7, the control group had the smallest wound size, followed by the HP group (p<0.001). On day 10, the HP group had the smallest wound size, while the DM group had the largest (p<0.001). Finally, on day 14, all groups demonstrated significant differences; the HP group had the smallest wound size, and the DM group had the largest (p<0.001) (**Table 4**, **Figure 6a**).

**Table 4:** Wound area measurement results (mean and SD) for each study group based on the treatment period.

Wound area (mm^2^)	Control	DM	Met	HP	p
Day 0	362.13 ± 8.51^a^	387.25 ± 8.08^c^	384.38 ± 10.82^bc^	377.12 ± 11.03^b^	**<0.001**
Day 3	269.25 ± 15.15^a^	290.12 ± 9.60^b^	279.88 ± 14.73^ab^	278.13 ± 12.03^ab^	**0.030**
Day 7	143.75 ± 8.41^a^	183.63 ± 7.27^c^	186.50 ± 12.10^c^	156.25 ± 13.68^b^	**<0.001**
Day 10	84.63 ± 11.03^b^	127.75 ± 7.52^c^	92.88 ± 7.94^b^	53.62 ± 8.04^a^	**<0.001**
Day 14	50.88 ± 6.98^b^	85.13 ± 7.20^d^	63.38 ± 6.87^c^	30.63 ± 6.61^a^	**<0.001**

P< 0.001 for all pairwise comparisons across experimental days within each group.

^a,b,c,d^Different superscript letters denote significant differences between the groups in each measurement time according to the post hoc test.

DM; Diabetes mellitus, Met; Metformin, HP; Hypericum perforatum, mm^2^; millimeters square.

Bold values indicate statistically significant differences (p<0.05).

The wound contraction percentages from day 0 to 14 were statistically significant across all groups (p<0.001). There was no significant difference in wound contraction rates between the groups on days 0-3 (p = 0.771). On days 0-7, the control and the HP groups showed higher contraction rates compared to the DM and Met groups (p<0.001). On days 0-10, the HP group exhibited the highest contraction rate, while the DM group had the lowest (p<0.001). By day 0-14, all groups showed significant differences, with the highest contraction in the HP group and the weakest in the DM group (p<0.001) (**Table 5**, **Figure 6b**).

**Table 5:** Percentage change (wound contraction rate) in wound diameter of the groups over the study period.

Wound contraction (%)	Control	DM	Met	HP	p
Days 0-3	25.64 ± 3.88	25.07 ± 2.23	27.10 ± 5.00	26.17 ± 4.27	0.771
Days 0-7	60.32 ± 1.68^a^	52.57 ± 1.77^b^	51.44 ± 3.56^b^	58.57 ± 3.31^a^	**<0.001**
Days 0-10	76.66 ± 2.68^b^	66.96 ± 2.58^c^	75.79 ± 2.50^b^	85.81 ± 1.85^a^	**<0.001**
Days 0-14	85.95 ± 1.88^b^	78.02 ± 1.68^d^	83.47 ± 2.09^c^	91.88 ± 1.73^a^	**<0.001**

P< 0.001 for all pairwise comparisons across experimental days within each group.

^a,b,c,d^Different superscript letters denote significant differences between the groups in each measurement time according to the post hoc test.

DM; Diabetes mellitus, Met; Metformin, HP; Hypericum perforatum.

Bold values indicate statistically significant differences (p<0.05).

There was no statistically significant difference among the groups in the collagen index, TGF-β percentage, and H scores (p = 0.118, p = 0.660, and p = 0.647). The fibroblast density scores of the DM group were significantly lower than those of the other groups (p=0.004). The hypertrophic index values of the HP group were the highest compared to those of the other groups (p = 0.003). Despite the statistical significance of this difference, the findings should be approached with caution owing to the limited sample size. When evaluating the total histopathological scores, the HP group had the highest score, followed by the control, the Met, and the DM groups, in that order (p = 0.002). TGF Beta was broadly expressed but at very low levels. Although the HP and control groups showed higher scores than the other groups, the differences were not statistically significant (**Table 6**).

**Table 6:** Comparison of histopathological assessments across the groups.

Histopathology	Control	DM	Met	HP	p
Fibroblast density	3499.50 ± 329.17^a^	2826.63 ± 478.59^b^	3362.63 ± 575.24^a^	3686.38 ± 367.67^a^	**0.004**
Hypertrophic index	2.05 ± 0.64^ab^	1.63 ± 0.38^b^	1.71 ± 0.29^b^	2.48 ± 0.40^a^	**0.003**
Collagen index	52.48 ± 7.92	51.68 ± 7.76	51.45 ± 5.66	58.97 ± 6.07	0.118
TGF Beta	34.73 ± 18.27	28.05 ± 13.31	27.66 ± 16.02	34.98 ± 14.28	0.660
H score	35.06 ± 18.56	27.83 ± 14.00	27.88 ± 16.32	35.21 ± 14.36	0.647
Total score	6.25 ± 1.28^ab^	4.75 ± 0.89^c^	5.50 ± 0.76^bc^	7.00 ± 1.20^a^	**0.002**

^a,b^Different superscript letters denote significant differences between the groups in each score (raw) according to the post hoc test.

DM; Diabetes mellitus, Met; Metformin, HP; Hypericum perforatum.

Bold values indicate statistically significant differences (p<0.05).

When the components that make up the SPOT score are examined in detail, no statistically significant differences were found between the groups regarding re-epithelization, keratinization, granulation tissue, remodeling, and scar elevation index (p > 0.999, p = 0.478, p > 0.999, p = 0.160, and p = 0.104). The only notable difference was observed in the epidermal thickness index, where all rats in the DM and 7 (87.5%) rats in the Met groups had a score of 1, which was significantly lower than that of the control and the HP groups (p = 0.032) (**Table 7**).

**Table 7:** Histopathological assessment of wound healing using the SPOT scoring system across groups.

SPOT scoring system	Control	DM	Met	HP	p
Re-epithelization, n (%)					
0	0 (0.0)	1 (12.5)	0 (0.0)	0 (0.0)	>0.999
1	7 (87.5)	7 (87.5)	8 (100)	7 (87.5)	
2	1 (12.5)	0 (0.0)	0 (0.0)	1 (12.5)	
Epidermal thickness index, n (%)					
1	3 (37.5)^a^	8 (100)^b^	7 (87.5)^b^	5 (62.5)^ab^	**0.032**
2	5 (62.5)^b^	0 (0.0)^a^	1 (12.5)^a^	3 (37.5)^ab^	
Keratinization, n (%)					
0	4 (50.0)	6 (75.0)	6 (75.0)	3 (37.5)	0.478
1	2 (25.0)	2 (25.0)	2 (25.0)	3 (37.5)	
2	2 (25.0)	0 (0.0)	0 (0.0)	2 (25.0)	
Granulation tissue, n (%)					
0	1 (12.5)	1 (12.5)	0 (0.0)	1 (12.5)	>0.999
1	7 (87.5)	7 (87.5)	8 (100)	7 (87.5)	
Remodeling, n (%)					
0	1 (12.5)	3 (37.5)	0 (0.0)	1 (12.5)	0.16
1	7 (87.5)	4 (50.0)	8 (100)	5 (62.5)	
2	0 (0.0)	1 (12.5)	0 (0.0)	2 (25.0)	
Scar elevation index, n (%)					
0	2 (25.0)	1 (12.5)	0 (0.0)	0 (0.0)	0.104
1	4 (50.0)	6 (75.0)	7 (87.5)	3 (37.5)	
2	2 (25.0)	1 (12.5)	1 (12.5)	5 (62.5)	

^a,b^Different superscript letters denote significant differences between the groups in each score (raw) according to the post hoc test.

DM; Diabetes mellitus, Met; Metformin, HP; Hypericum perforatum.

SPOT; Stellenbosch University, Polish Academy of Sciences, Obatala Sciences, and the University of Texas Southwestern.

Bold values indicate statistically significant differences (p<0.05).

## 4 Discussion

This study investigated the effects of HP and metformin on wound healing in diabetic rats, focusing on the gross wound healing rate, histopathological, and immunohistochemical parameters. The data indicate that both active ingredients, especially HP, have beneficial effects on wound healing.

Wound healing involves three overlapping phases: inflammation, proliferation, and remodeling. During inflammation, cytokines and growth factors are released at high levels, attracting inflammatory cells to the wound site. In the proliferation phase, fibroblasts are activated, and collagen is produced, while in the remodeling phase, collagen fibers are reorganized to support tissue maturation. Because of the biochemical and cellular differences specific to each phase, the timing of measurements is important (30). In our study, no significant differences were observed between groups in TGF-β staining percentage, H-score, and collagen density. The lack of substantial differences in these parameters may be due to the small sample size, as well as the complex biological processes involved in wound healing and the various mechanisms active at different stages (31). For instance, TGF-β reaches its peak during the inflammation phase and remains elevated during the proliferation phase, where it encourages fibroblast activation and collagen synthesis, but its levels decline during the remodeling phase (32). Therefore, measurements obtained at a single time point may be inadequate to accurately represent the true biological distinctions among groups. Specifically, the variable nature of TGF-β, together with the limited sample size, has impeded the ability to identify statistical differences. This observation underscores the necessity for further analyses involving extended follow-up periods and various phases in future studies. A similar pattern applies to collagen accumulation and organization.

The observation that the metformin group demonstrated lower outcomes compared to the HP group regarding fibroblast density and hypertrophic index implies that the influence of metformin on wound healing may primarily pertain to inflammation regulation and tissue remodeling (14). The fibroblast proliferation and epidermal healing-promoting effects exhibited by the HP group align with existing literature (33). These findings substantiate the concept that wound healing constitutes a multifaceted process involving various mechanisms.

In the SPOT histopathological evaluation system, a significant difference was observed solely in the epidermal thickness index, suggesting that the HP group demonstrated greater efficacy in epidermal repair. Although no significant differences were identified among other subparameters, the significance of the overall score underscores that composite scores possess greater statistical power than individual parameters, allowing small differences to attain significance when aggregated. Furthermore, the lack of significant findings in the subparameters may be attributable to the limited sample size or the differential impact of parameters at various stages of wound healing (27).

Statistically significant increases in blood glucose levels were observed in all three groups (DM, met, HP) after STZ administration. Although STZ was not given, the rise in blood glucose in the control group was not considered to be within diabetic limits and was thought to be related to surgical stress, which aligns with the literature (34).

Although primarily observed in patients with chronic conditions, wound healing issues are also frequently encountered in patients with DM during the acute phase due to elevated blood glucose levels. It is well documented that over 100 physiological factors, including impaired macrophage function, altered growth factor production, inadequate collagen accumulation, compromised granulation tissue quality, and decreased fibroblast proliferation and migration, are affected by DM (1). Wound healing complications are increasingly being reported in pediatric patients with DM. Given that the incidence of type 1 DM is rising among children under the age of 4, it is evident that a greater number of children will face risks of complications associated with chronic DM over time (4). At initial diagnosis, it requires time for blood glucose levels to stabilize; consequently, children often need to perform fingertip pricks 10–12 times daily to monitor their blood glucose. Furthermore, with technological advancements, the utilization of continuous glucose monitoring sensors and insulin pumps is on the rise, and issues related to skin and wounds associated with these devices are becoming more prevalent (5).

Lipodystrophy is one of the most significant dermatological issues seen in areas receiving insulin injections and can lead to serious skin problems like chronic suppurative infections (35). This condition, which manifests primarily as either lipohypertrophy or, less frequently, lipoatrophy, is predominantly noticed in sites of chronic insulin administration. Given that patients, particularly children, often have injections performed in the same location due to cosmetic considerations, the probability of developing this complication is elevated (36).

HP, which has been extensively utilized as a traditional remedy in Europe and the Middle East for numerous years and is readily available in nature, is employed in the treatment of various diseases. Its applications encompass depression, hemorrhoids, skin ulcers, and burns (37). Certain experimental investigations have demonstrated that it reduces the duration of inflammation, enhances fibroblast migration, and exerts favorable effects on collagen synthesis and accumulation (7, 37). Beyond experimental research, some clinical studies have also examined the influence of HP on wound healing (11, 38). The therapeutic efficacy of HP has been further explored in pediatric burn cases, with reports indicating that it facilitates the healing process by alleviating pain and inflammation (12). Additionally, some research has focused on the impact of HP on DM regulation when administered systemically, with findings suggesting that orally administered HP extract effectively controls blood glucose levels (6, 39). Two distinct formulations have been developed for the topical and oral applications of HP. The ethanol extract, which is rich in hydrophilic constituents, is preferred for oral administration and has been employed in treating conditions such as depression. Conversely, the olive oil extract is more lipophilic, and it has been demonstrated that the effects of hypericin and hyperforin—the active constituents of HP—are potentiated by the polyphenols present in olive oil. The olive oil extract is predominantly used for topical application, with known minimal systemic absorption (10, 40). In our study, the olive oil extract of HP was utilized, and in accordance with the literature, rats treated with HP exhibited statistically significant improvements in wound size, contraction rate, and overall histopathological scores compared to other groups, despite having uncontrolled DM. The elevated hypertrophic index values observed in the HP group are noteworthy; however, given the biological variability and the limited sample size, prudence is advised when considering the clinical significance of this observation.

Metformin, a first-line pharmacological agent for the treatment of type 2 DM globally, is a biguanide compound that facilitates wound healing through the enhancement of angiogenesis, re-epithelialization, and collagen deposition (19). Several studies have also demonstrated that it diminishes the expression of metalloproteinases (MMPs), which serve as pro-inflammatory mediators (41). Despite its widespread usage, there is a paucity of research investigating the local effects of metformin on the wound healing process in the literature. While most studies have reported positive effects on wound repair (14, 42, 43), others have indicated that topical and systemic application of metformin, especially following burn injuries, may exert adverse effects by inhibiting cell proliferation (17, 18). It has been suggested that the detrimental impact associated with this antiproliferative property post-burn injury could potentially yield beneficial outcomes in scar formation during the chronic phase in burn patients (44). Therefore, it is understood that metformin’s effects on wound healing can vary depending on how it is administered, the dose, and the type of injury. This highlights the need for careful dose optimization and proper indication selection for future clinical use of the drug. Additionally, the differing results seen in various experimental models suggest that the dual effects of metformin should be further explored through more detailed mechanistic studies. Although various dosages of topical metformin have been documented in scholarly literature (14, 42), it has been noted that the application of a 10% gel is more standardized. Furthermore, the gel formulation demonstrates significant localized effects even with a single daily application to the wound (22). Upon analysis of the data from our study, it was observed that the wound size, wound contraction rate, and overall histopathological scores in diabetic rats treated with 10% metformin gel were comparable to those in the non-diabetic control group. These findings, which align with existing literature, provide optimism that the adverse effects of uncontrolled diabetes on wound healing can be mitigated through the localized application of metformin.

Our study has certain limitations. First, the small sample size was limited by ethical considerations. Additionally, a 14-day assessment of wound healing only captures the initial phase of tissue remodeling. Furthermore, the anatomical differences in skin structure between humans and rats mean that further research involving human subjects is necessary to confirm our findings. Moreover, additional studies are needed to thoroughly evaluate parameters such as the local absorption rate and the participation of systemic circulation in topically applied HP and metformin. The levels of hyperforin and hypericin in the Hypericum perforatum extract were not measured, which limits reproducibility due to lack of standardization. Also, our study does not fully reflect wound healing mechanisms, as molecular markers like vascular endothelial growth factors (VEGF), cytokines, and oxidative stress markers were not assessed. Future research focusing on these additional molecular parameters may offer more comprehensive insights into how HP and metformin contribute to wound healing. Furthermore, this study examined the effects of metformin and HP separately. Future studies exploring the potential synergistic effects of combining HP and metformin could lead to new treatment strategies for diabetic wound healing.

In conclusion, this study is the first in the existing literature to examine and compare the effects of HP and metformin on wound healing in diabetic rats within a single research framework. The data collected indicate that locally applied HP in uncontrolled DM rats showed better wound healing scores compared to the non-diabetic control group. Meanwhile, metformin produced statistically significant wound healing scores in the DM group, and the results were similar to those of the control group. Given that HP and metformin are easily accessible substances, we suggest that further research could help develop cost-effective treatments for wound healing in DM patients and address dermatological issues seen at insulin injection sites.

## Data Availability

The original contributions presented in the study are included in the article/supplementary material. Further inquiries can be directed to the corresponding author.
